# Remedy for Inflamed and Sore Mouths, Caused by Wearing Artificial Teeth on Rubber Plates

**Published:** 1883-08

**Authors:** D. G. Harkins

**Affiliations:** Holyoke, Mass.


ARTICLE VI.

### REMEDY FOR INFLAMED AND SORE MOUTHS CAUSED BY WEARING ARTIFICIAL TEETH ON RUBBER PLATES.

BY D. G. HARKINS, D. D. S., HOLYOKE, MASS.

I have recently noticed in reading the various Dental Journals, that there is considerable discussion relative to the injurious effects of wearing artificial teeth on vulcanite rubber plates. As I have had considerable experience in this direction, and not having seen or heard of any remedy or cure, I would suggest a method which has been attended by very gratifying results. I feel convinced that it will, prove as successful with others as it has with me. My course of procedure is as follows:

After taking the impression and constructing the model, I obtain the bite; (articulation) and form my trial plate, but instead of the gutta-percha usually used, I substitute vulcanite gutta-percha. I then select my teeth, grind and adjust them on the vulcanite gutta-percha trial plate, which becomes part of the permanent plate. This material does not expand or contract, thus avoiding breaking of teeth; neither does food collect over it causing irritation of the gums. It is, besides, free from the deleterious effects and objections of rubber, being manufactured by an entirely different process. It is also intended to cover the palatine surface of the mouth. The objections raised against gutta-percha heretofore was its tendency to form small air pits. This may be obviated by heating the plaster model over a laboratory lamp or stove. This precaution will absorb all the moisture in the plaster. I then cover or line the model with tin foil on the part of plate intended to cover the roof of the mouth. I afterwards cut a piece of sheet rubber sufficient to cover the lingual surface; this I heat and apply directly over the vulcanite gutta-percha, and pack small pieces of the rubber over the labial surface. When this has been accomplished, I unite both sections of the flask together, and put into the vulcanizer where it is kept at 320 degrees Fr. for about 45 minutes. A little experience will determine the exact time. After unflasking, and the tin foil removed, the palatine surface will present a bright polished appearance on the vulcanite gutta-percha portion of plate, which will not irritate or cause disease of the mouth, but which will be a better fitting and satisfactory denture in every respect than by the old method. It will be observed that by this process all the advantages of the rubber is obtained on the lingual surface, where it will have no injurious effect, together with the benefit of gutta-percha in one plate for artificial teeth.

I have been using the combination of vulcanite gutta-percha and rubber plates for over a year; and I have not as yet met with the first failure. Some who had been wearing upper rubber plates complained of the mercurial effects on the mucous membrane of the mouth—a soreness and burning feeling, I substituted a combination of vulcanite gutta-percha and rubber, and since then the irritation and soreness (which some medical men attribute to the Sulphuret of Mercury contained in the rubber plate) has entirely disappeared.

Those who have not tried this new method (I believe it is new) I would earnestly ask a trial of it, believing that it will meet with approval.

Why should we discard vulcanite as a base for the insertion of artificial teeth when it has proved such a boon to the human family? I have been taught in the colleges the different methods of mounting artificial teeth on the materials generally employed, such as gold plate, silver plate, platina plate, continuous gum work, cheap plastic plate etc., and I confidently assert that in the use of all these, I have failed to secure so accurate a fitting set of artificial teeth, so satisfactory to myself and patients as that perfected by the use of vulcanite plates.

